# Nursing in the United States

**Published:** 1916-05-20

**Authors:** 


### THE MATRONS' AND SISTERS' DEPARTMENT

### III. NURSING IN THE UNITED STATES.

### STATE REGISTRATION IN AMERICA.

We are confident the College of Nursing will do much to co-ordinate nurse training and practice both in Great Britain and the United States. Dr. Hurd concludes his letter with the following interesting facts based upon practical experience.

#### Shortage of Probationers for Training.

Question: *Will you kindly give me information as to the shortage or increase in the number of women seeking training as certificated nurses in the United States?*

Dr. Hurd's Answer: In the matter of the shortage of nurses other factors enter besides compulsory registration. In my judgment, the lack of protection to the rights of better nurses has been an important factor in the shortage of applicants. The well-educated nurse who has spent three years in securing her education and is fitted for work ought not to be compelled to compete with a nurse from a correspondence school, who has received her training by correspondence in three months without practical experience and at an expense of only 26 dollars. The shortage of nurses has also arisen to some extent from the fact that many schools compel their nurses to work too many hours with insufficient food, unhealthy apartments, and unsatisfactory educational advantages.

#### The Training School as an Educator.

Question: *What effect have recent developments in the United States had upon the improved education and efficiency of nurses?*

Dr. Hurd's Answer: Where the training school has been established as an educational school, rather than as a convenience for the hospital to save money and to avoid the necessary expense of ward-maids, there has not been much difficulty in getting a sufficient number of probationer-nurses.

#### The General Effect of Registration.

Question: *What has been the general effect of registration in the United States?*

Dr. Hurd's Answer: The general effect of registration has been to make nurses better educated and more efficient, and to give an *esprit de corps* to their profession.

From a recent statement published by Dr. Hurd, it appears that in the United States school nurses, Red Cross nurses, visiting nurses, and Army nurses are universally chosen from among those who have had proper education and adequate training. In certain States, although the registration laws have been weak, they have still had the effect of raising the general standard of education. Thus the smaller schools have felt encouraged to affiliate themselves with others possessing better facilities, and the State Board have given valuable assistance to the smaller schools by stimulating them to increase the length and scope of their curriculum. As a result of this action the outlook in the United States in regard to such schools is encouraging. On this experience Dr. Hurd bases an urgent appeal to all professional and other persons interested in nurses to use their utmost efforts to teach and enforce efficiency throughout the nurse-training schools of the United States.

